# A misplacement of a ureteral stent into the abdominal aorta: a case report of a rare retrograde ureteral stenting complication

**DOI:** 10.1186/s12905-022-02049-6

**Published:** 2022-11-18

**Authors:** Nebojsa Prijovic, Bojan Cegar, Vladimir Cvetic, Veljko Santric, Branko Stankovic, Jovan Radojevic

**Affiliations:** 1 Clinic of Urology, University Clinical Center of Serbia, Resavska 51, Belgrade, Serbia; 2 Faculty of Medicine, University of Belgrade, Dr Subotica 8, Belgrade, Serbia; 3 Clinic for Vascular and Endovascular Surgery, University Clinical Center of Serbia, Dr Koste Todorovica 8, Belgrade, Serbia; 4 General Hospital “Dr Radivoj Simonovic” Sombor, Vojvodjanska 75, Sombor, Serbia

**Keywords:** Complication, Hydronephrosis, Ureteral stent, Abdominal aorta

## Abstract

**Background:**

Cervical cancer is often associated with malignant ureteral obstruction and consequent hydronephrosis. Hydronephrosis caused in this way can be resolved by placing ureteral stents or performing a percutaneous nephrostomy. Complications that may occur during the retrograde ureteral stent placement are usually mild, but serious complications such as stent migration into the cardiovascular system are also possible. Here we present an unusual case where a ureteral stent entered the abdominal aorta during the cystoscopic ureteral stenting, which was resolved by a cystoscopic stent removal kept in check by endovascular catheters.

**Case presentations:**

The 48-year-old female patient was treated in the regional secondary healthcare facility due to bilateral hydronephrosis caused by cervical cancer. The patient had bilateral percutaneous nephrostomies and ureteral stents. Due to the calcification of the left ureteral stent, an urethrorenoscopy with lithotripsy of the calculus in the left ureter was performed in the regional secondary healthcare facility, and the ureteral stent was cystoscopically replaced. The control radiography of the urinary tract showed a misplacement of the left ureteral stent, and a computed tomography showed that the stent was located in the abdominal aorta. The patient was referred to the University Clinical Center of Serbia, where a ureteral stent was cystoscopically removed from the abdominal aorta under the control of endovascular catheters. The patient was in good general condition at all times, with no signs of bleeding, and she was discharged from the hospital on the fourth postoperative day.

**Conclusions:**

The migration of a ureteral stent into the abdominal aorta and the cardiovascular system in general is a rare type of ureteral stenting complication whose treatment requires a multidisciplinary approach. In order to prevent such complications, it is necessary to strictly adhere to the indications for the ureteral stent placement in the case of malignant ureteral obstruction. Also, this procedure should be performed according to the current guidelines and controlled by an X-ray or ultrasound.

## Background

Cervical cancer can frequently cause a malignant ureteral obstruction in any stage of the disease and can then lead to hydronephrosis [1]. In such cases, hydronephrosis can be treated by a ureteral stent placement or by percutaneous nephrostomy (PCN). Ureteral stent placement is considered appropriate for the initial treatment of hydronephrosis; however, in numerous cases this treatment can be either inapplicable or ineffective, in which case, the placement of PCN is required [1–3]. In certain selected cases, the choice of treatment could depend on the patient’s or urologist’s preferences. Ureteral stenting, regardless of whether it is performed via the antegrade or retrograde method, could cause numerous complications, such as hematuria, pain, infection, calcification or fragmentation [4]. Complications are usually mild, but could at times be severe and difficult to treat, such as stent migration into the cardiovascular system or even the pleural cavity [5–8]. In such cases, the treatment of these complications is complex, and requires a multidisciplinary approach.

We present a rare case where a ureteral stent entered the abdominal aorta during the cystoscopic ureteral stenting, which was resolved by a cystoscopic stent removal kept in check by endovascular catheters without the need for embolization.

## Case presentation

A 48-year-old female patient was treated for 6 years by a urologist in a regional secondary healthcare facility for bilateral hydronephrosis, caused by cervical cancer treated with radical radiotherapy and chemotherapy. The treatment of bilateral hydronephrosis was performed with the bilateral placement of ureteral stents and PCNs which were regularly and periodically replaced. Due to an unsuccessful cystoscopic replacement of the ureteral stent on the left side 1 month prior to that, and due to calcification around the stent in the left ureter, the urologist in charge indicated that ureteroscopy with lithotripsy of the calculus in the left ureter should be performed. The patient underwent a procedure of the left side ureteroscopy with ultrasonic lithotripsy of the 6 mm ureteral calculus, positioned approximately 6 cm from orifice, under general anesthesia, in the designated healthcare facility. Due to technical reasons, the already existing ureteral stent on the left side was removed cystoscopically, and another ureteral stent was inserted into the left ureter.

Following the procedure, a three-way urinary catheter was placed into the bladder, and a slightly red urine was formed. The patient coped quite well, she was in good general postoperative condition, without pain, hemodynamically stable, with normal heart rate, afebrile. In the first 10 minutes following the procedure, there was a mild macroscopic haematuria, after which her urine became completely macroscopically clear.

**Fig. 1:**
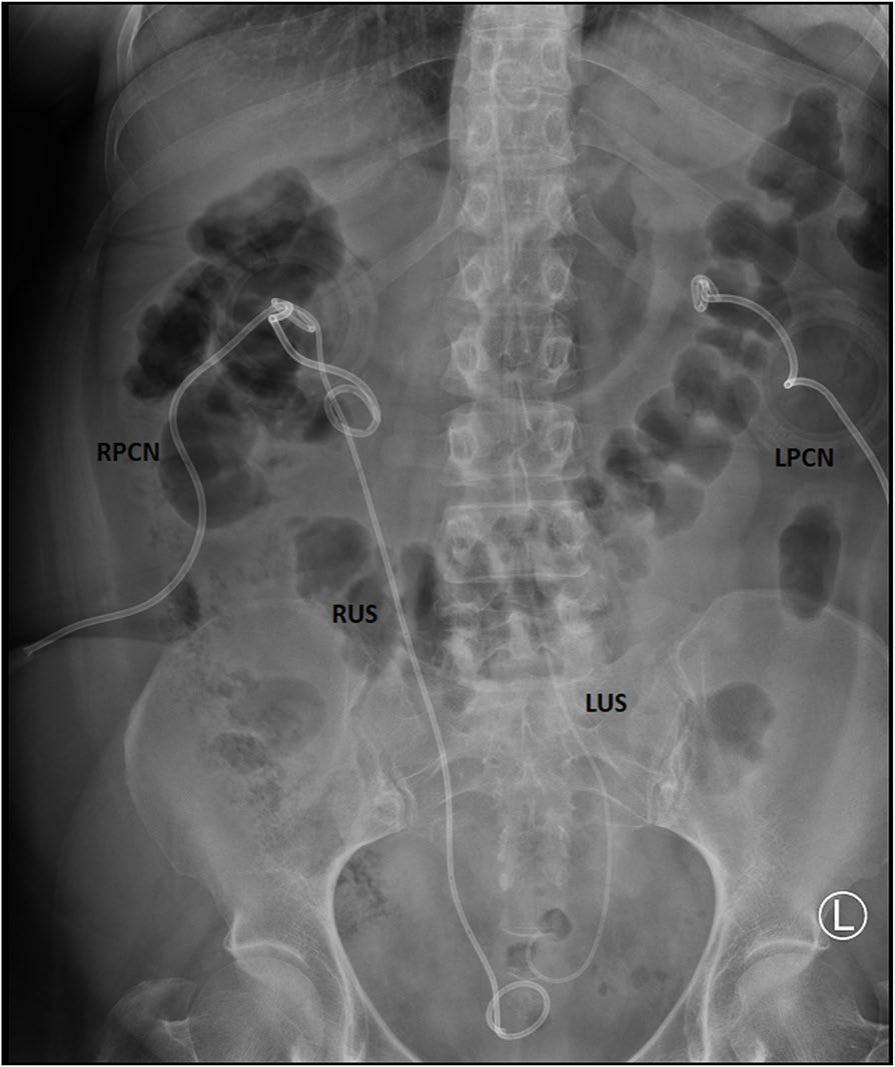
Plain radiography of the urinary tract shows a malposition of the left ureteral stent. LPCN left percutaneous nephrostomy; LUS left ureteral stent; RPCN right percutaneous nephrostomy; RUS right ureteral stent

**Fig. 2:**
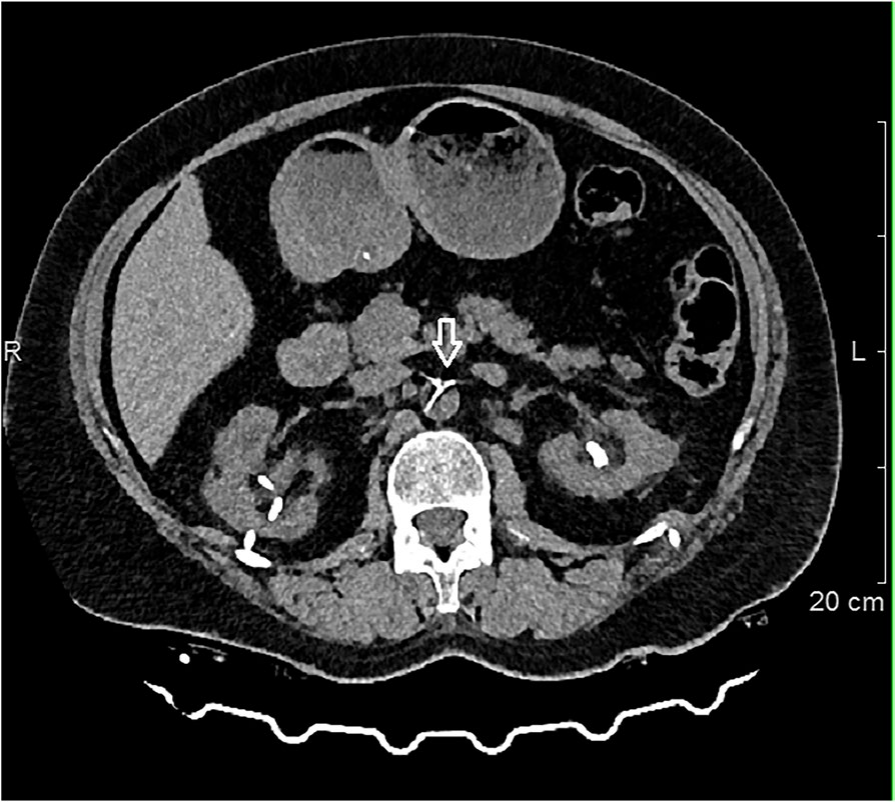
Computed tomography of the abdomen and pelvis shows the position of the ureteral stent in the abdominal aorta (arrow), transverse plane

**Fig. 3:**
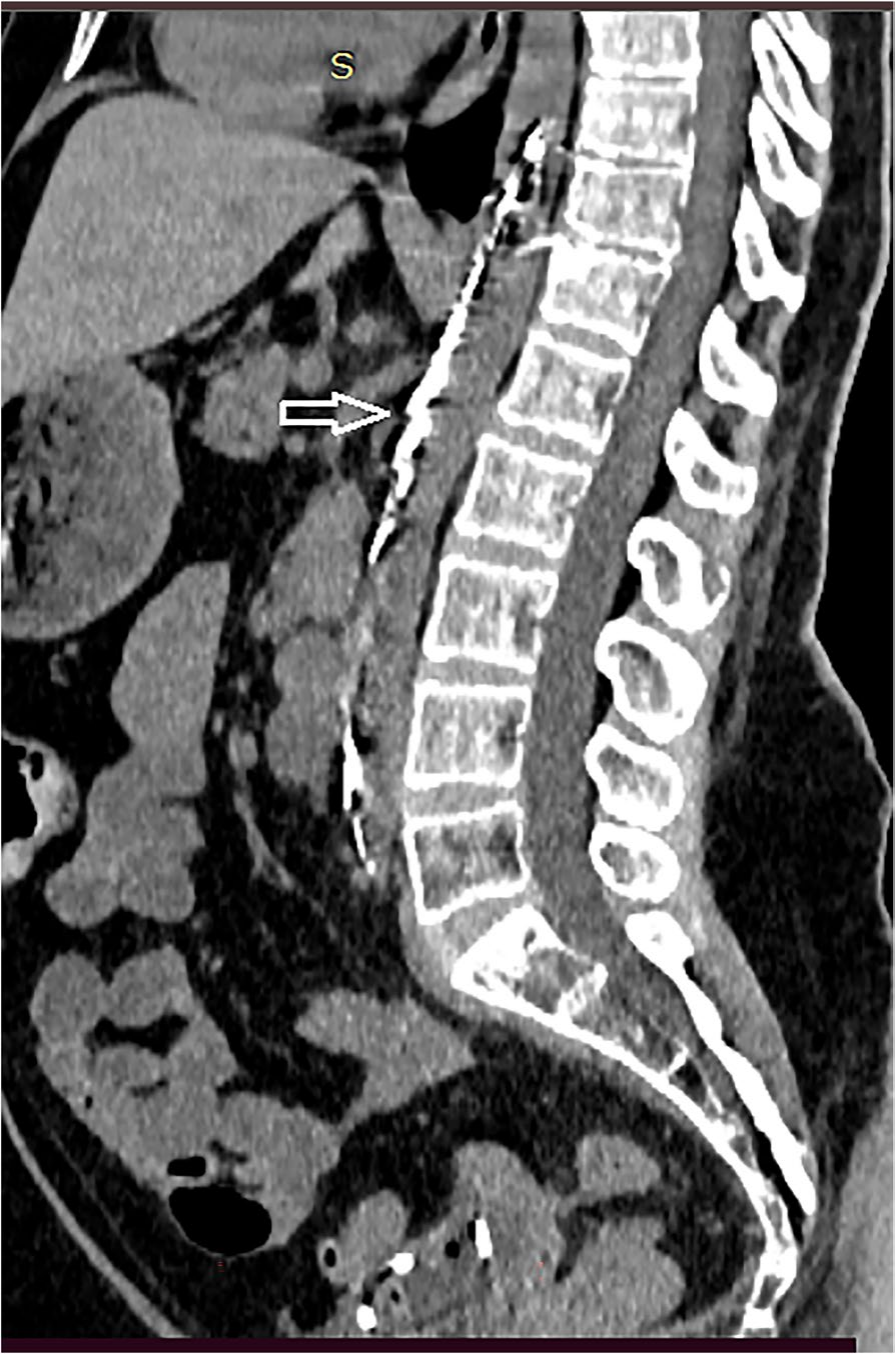
Computed tomography of the abdomen and pelvis shows the position of the ureteral stent in the abdominal aorta (arrow), sagittal plane

On the same day, radiography of the urinary tract was performed, when it was seen that the ureteral stent on the left side was dislocated, and that it was in summation with the spine (Fig. 1). An urgent computerized tomography of the abdomen and pelvis with contrast was performed, which showed that the ureteral stent went through the left ureter in the length of 65 mm from the orifice, piercing through the left internal iliac artery at approximately 35 mm from the bifurcation of the left common iliac artery, continuing through the abdominal aorta where its tip was at the level of the 11th thoracic vertebrae (Figs. 2 and 3). Neither the contrast extravasation nor the presence of a hematoma was observed. The patient was referred to the Clinic of Urology of the University Clinical Center of Serbia that same day for further multidisciplinary treatment.

Following the admission to the Clinic of Urology, and due to high risk from potential complications, the patient was initially admitted to the intensive care unit. At the admission, the patient was conscious, communicative, without pain, afebrile (36,5 °C), with normal vital signs (blood pressure 110/80 mmHg, heart rate 70–80/min). The physical examination revealed the abdomen to be in line with the chest cavity, without defacement, insensitive to pain during palpation, bilateral PCN were present with clear urine. Laboratory investigations at the admission indicated the following: red blood cells 2,32 × 10^12^/L, hemoglobin 101 g/L, hematocrit 0,321 L/L, white blood cells 9,9 × 10^9^/L, platelets 151 × 10^9^/L.

**Fig. 4:**
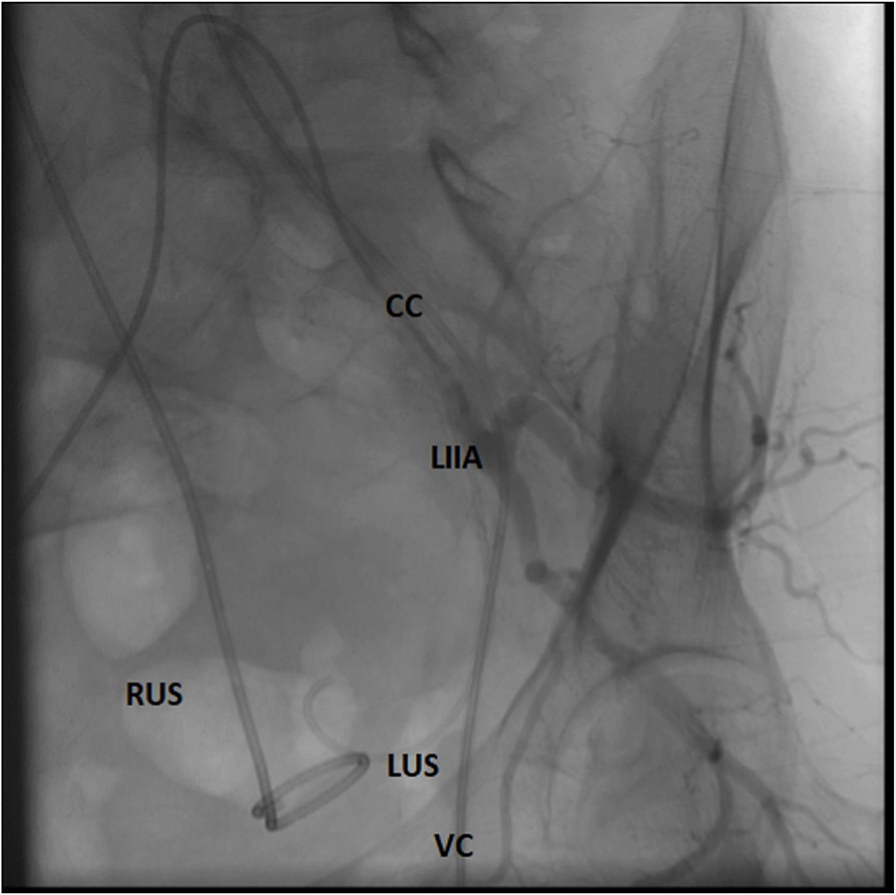
Endovascular catheters and ureteral stents and their position at the beginning of the procedure. CC Cobra 5F catheter; VC Vertebral catheter; LIIA left internal iliac artery; LUS left ureteral stent; RUS right ureteral stent

**Fig. 5:**
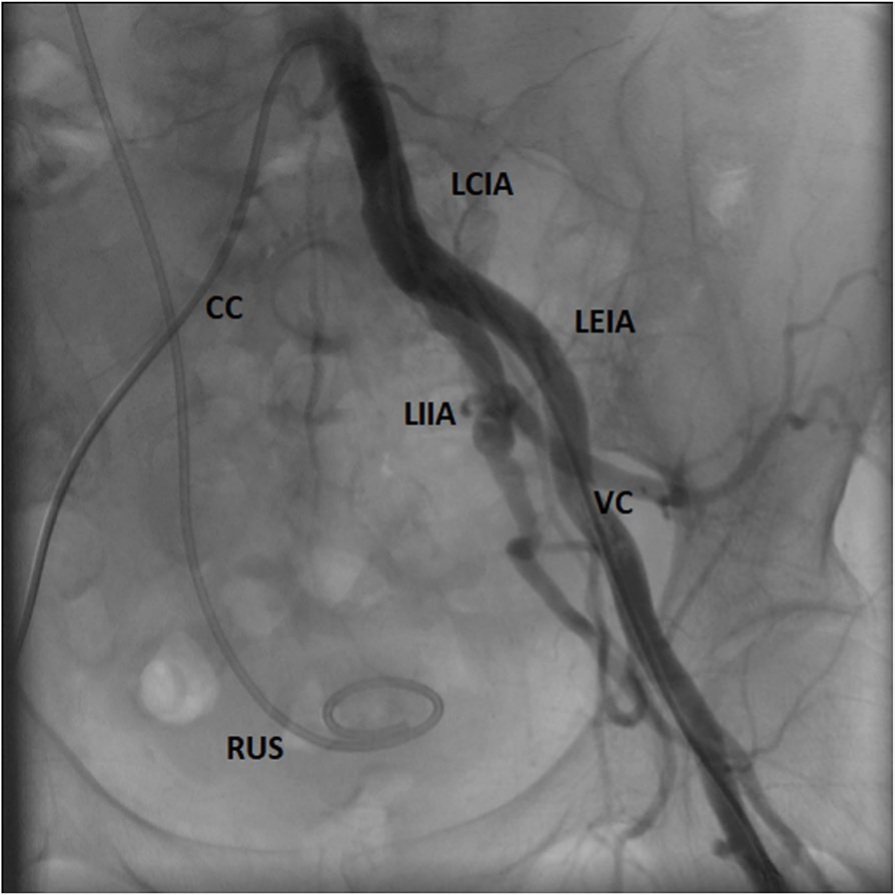
Angiography at the end of the procedure shows no contrast extravasation. CC Cobra 5F catheter; VC Vertebral catheter; LCIA left common iliac artery; LIIA left internal iliac artery; LEIA left external iliac artery; RUS right ureteral stent

The following day, the patient was transferred to the Clinic for vascular and endovascular surgery, so that the cystoscopic extraction of the ureteral stent could be performed. After the adequate preparation of the surgical area and under the local anesthesia and fluoroscopic monitoring, 5F Cobra catheter was inserted through the right femoral artery into the patient’s left internal iliac artery, at the place where the ureteral stent penetrated the aortic wall. A control Vertebral catheter was inserted through the left femoral artery into the left common iliac artery. Aortography was performed, and the position of the ureteral stent indicated that it penetrated the aortic wall, and that its proximal end was in the aorta at the level of the thoracoabdominal junction (Fig. 4). A rigid cystoscope was inserted into the bladder. Cystoscopic pincers were used to grab the ureteral stent in the left orifice, the stent was then pulled out and then extracted from the aorta, common iliac artery and bladder under the fluoroscopic control. Following the procedure, angiography indicated that there was no extravasation of contrast from the aorta and the left common and internal iliac artery (Fig. 5). Therefore, there was no need for embolization. The patient successfully underwent the procedure, and was then transferred back to the Clinic of Urology that same day. During hospitalization, the patient stated she felt well, she was haemodynamically stable, without visible signs of bleeding, and without reduced levels of hemoglobin. The patient was discharged from the Clinic of Urology on the fourth postoperative day with stable vital signs.

## Discussion and conclusions

Hydronephrosis is frequently developed in patients suffering from cervical cancer, and is commonly associated with more advanced disease stages [1]. It occurs as a consequence of a tumor or lymph nodes growth, inflammation or fibrosis in pelvis, which results in pain occurrence, infection, or renal malfunction due to obstruction. Furthermore, it is considered to be an indicator of a bad prognosis in such patients [9].

There are several hydronephrosis treatment models for patients with cervical cancer and the placement of ureteral stents is considered the primary choice for the initial treatment of ureter obstruction [1, 2]. The treatment of hydronephrosis can be performed with the insertion of PCNs in cases when the placement or replacement of ureteral stents is not possible or considered inefficient, such as in the case of advanced hydronephrosis, renal malfunction, infection or pain occurrence [3]. The studies conducted so far have shown that in 16–58% of patients with malignant ureteral obstruction, the placement of ureteral stents is unsuccessful [3, 10]. In their study from 2004, Ku et al. have shown that hydronephrosis treatment outcomes, where ureteral stents or PCNs were used, were similar [11]. Results from a more recent study by Netsch et al. have shown that there is no difference in the survival rate and complication occurrence among patients with malignant ureteral obstruction who were treated by a ureteral stent placement and a PCN placement [12]. When deciding on the kind of hydronephrosis treatment, significant factors are the patient’s and urologist’s preferences [13, 14], which in our case was the decisive factor, as both sides were for the PCN removal.

Ureteral stents have been used since 1967 in the treatment of ureteral obstruction caused by calculosis, stenosis, trauma or tumor compression, regardless of whether they are used as a temporary or permanent solution [15]. The placement of ureteral stents can be antegrade or retrograde. In this case, a ureteral catheter is placed using a retrograde approach with the aim of treating hydronephrosis caused by cervical cancer. One of the complications connected with the ureteral stent placement is calcification which can occur around the stent [16], which is exactly what happened in this case, and which demanded endolithotripsy of the calculus and a stent replacement. Furthermore, complications may include a stent misplacement and migration [16]. In the case of our patient, during the cystoscopic placement, the stent migrated from the left ureter to the left iliac artery and to the abdominal aorta. During the retrograde stent placement, an X-ray monitoring is advised, but in our case, as in the majority of such cases, an X-ray was not possible, therefore the control radiography was done after the procedure had been performed.

The removal of ureteral stents from the circulatory or vascular system requires a multidisciplinary approach, and today it can be performed with an open, laparoscopic or endovascular approach [5–7]. In the case of our patient, the ureteral stent migrated into the abdominal aorta thus penetrating the ureter wall as well as the wall of the iliac artery which were damaged and fragile because of radiotherapy, frequent stent replacements, calculosis and ureterorenoscopy. Additionally, the urologist did not strictly follow the procedure of the ureteral stent placement that should be followed up by an X-ray, and the choice of the ureteral stenting in this case of malignant obstruction is questionable.

Until now, several cases of ureteral stent migration into the vein blood vessels, right ventricle and atrium or pulmonary arteries have been described [5–7, 17], but we have found only one case of stent migration into the abdominal aorta [18]. In that case, the stent migrated from the ureter which was damaged during laparoscopic hysterectomy. The stent was cystoscopically removed with the placement of endovascular catheters and X-ray monitoring. During the procedure, transcatheter super-selective embolization of the inferior gluteal artery was performed, at the place where the ureteral stent entered the bloodstream. In our case, after the removal of the ureteral stent, there were no signs of contrast extravasation from the blood vessels, and thus there was no need for embolization.

In order to prevent ureteral stent migrations, it should first be considered if there are any indications for the placement of the ureteral stent or PCN. When a urologist decides to perform a ureteral stent placement, and when the circumstances and conditions allow it, the procedure should be performed according to the existing guidelines. It is advised that the procedure be monitored by ultrasound or an X-ray. After the procedure, it is necessary to monitor the patient’s symptoms, as the symptoms and other signs can indicate that there may be complications. Furthermore, it is necessary to perform a radiographic evaluation of the stent position following the intervention. In the case of a stent misplacement or migration, the patient should be immediately treated, and a stent migration into the vascular or circulatory system requires a multidisciplinary approach.

## Data Availability

The datasets used and analyzed during the current study are available from the corresponding author on reasonable request.

## Abbreviation

PCN: Percutaneous nephrostomy
